# Crosstalk Between Peripheral Innervation and Pancreatic Ductal Adenocarcinoma

**DOI:** 10.1007/s12264-023-01082-1

**Published:** 2023-06-22

**Authors:** Bo Ni, Yiqing Yin, Zekun Li, Junjin Wang, Xiuchao Wang, Kaiyuan Wang

**Affiliations:** 1 Department of Pancreatic Cancer, Tianjin Medical University Cancer Institute and Hospital, National Clinical Research Center for Cancer, Key Laboratory of Cancer Prevention and Therapy, Tianjin’s Clinical Research Center for Cancer, Tianjin, 300060 China; 2 Department of Anesthesiology, Tianjin Medical University Cancer Institute and Hospital, National Clinical Research Center for Cancer, Key Laboratory of Cancer Prevention and Therapy, Tianjin’s Clinical Research Center for Cancer, Tianjin, 300060 China

**Keywords:** PDAC, Cancer-neuroscience, Peripheral innervation, Pain

## Abstract

Pancreatic ductal adenocarcinoma (PDAC) is a highly aggressive lethal malignancy, characterized by late diagnosis, aggressive growth, and therapy resistance, leading to a poor overall prognosis. Emerging evidence shows that the peripheral nerve is an important non-tumor component in the tumor microenvironment that regulates tumor growth and immune escape. The crosstalk between the neuronal system and PDAC has become a hot research topic that may provide novel mechanisms underlying tumor progression and further uncover promising therapeutic targets. In this review, we highlight the mechanisms of perineural invasion and the role of various types of tumor innervation in the progression of PDAC, summarize the potential signaling pathways modulating the neuronal-cancer interaction, and discuss the current and future therapeutic possibilities for this condition.

## Introduction

The tumor microenvironment (TME) is a complex acidic environment consisting of tumor and non-tumor cell types [1, 2], which plays a crucial role in the development and progression of tumors [3]. As an important part of the non-tumor element in the TME, the role and mechanism of tumor innervation have been increasingly investigated in various tumors including lung cancer [4, 5], melanoma [6–12], thyroid cancer [13–15], prostate cancer [16–21], breast cancer [22–25], ovarian carcinoma [26–28], head and neck cancer [29–33], gastric cancer [34–43], and pancreatic cancer [44–47], among others (Table 1). The crosstalk between the nervous system and cancer has favored the establishment of an interdisciplinary field—cancer neuroscience—and may provide additional potential therapeutic strategies.

**Table 1:** Peripheral innervation in different cancers with the role and potential therapeutic target

Cancer type	Sympathetic innervation	Parasympathetic innervation	Sensory innervation	Therapeutic targets
Lung cancer	Contributes to neovascularization [5]	Promotes cancer cell proliferation and invasion [4]	Evokes metastatic bone pain [137]	Muscarinic and nicotinic receptors [170, 171] TRPV1 [137]
Melanoma	Promotes tumor growth [7, 8] Promotes proliferation and tumor angiogenesis [10]	Infiltration and metastasis [9, 11]	Inhibits leukocyte recruitment, increases lymphoid and myeloid immunosuppressive cells [6, 12, 131]	Chemical sympathectomy [7] Beta-adrenergic receptors and glucocorticoid receptor [8]
Thyroid cancer	Related to PNI [13]	Promotes self-renewal and immune escape of thyroid cancer cells [14]	Inhibits tumor migration, invasion and adhesion [15]	None
Prostate cancer	Promotes tumor migration and metastasis [16–18] Promotes angiogenesis [20]	Induces tumor invasion and metastasis [17, 21]	Related to PNI [19]	Beta-adrenergic receptors [16, 17] Muscarinic acetylcholine receptors [17]
Breast cancer	Accelerates growth and progress [22, 24] Promotes lung metastatic colonization by circulating breast cancer cells [23]	Decreases PD-1 and PD-L1 expression [22]	Reduces adrenal metastases [25]	Beta-adrenergic receptors [112]
Head and neck cancer	Promotes survival and proliferation [30] Promotes tumor migration and metastasis [32]	Induces cell survival and cisplatin resistance [31]	Associated with tumorigenesis and immunosuppression [29] Promotes proliferation and migration and cytoprotective autophagy [33, 132]	Beta-adrenergic receptors [30, 32]
Ovarian carcinoma	Promotes tumor growth and angiogenesis [26, 172]	Promotes growth and/or proliferation [27]	Promotes proliferation [28]	Beta-adrenergic receptors [26]
Gastric cancer	Promotes epithelial-mesenchymal transition [34–37] Up-regulation of MMP-7 levels [38]	Promotes tumorigenesis [39] Promotes proliferation [40], invasion, and migration [41, 128]	Promotes tumor progression [42, 43, 173]	Beta-adrenergic receptors [38] Muscarinic acetylcholine receptors [40]
PDAC	Increases cancer growth [45, 46, 100]	Promotes an immunosuppressive microenvironment [125] Tumor budding [123]	Prevents neurogenic inflammation and delays tumor formation [44, 47]	Beta-adrenergic receptors [45, 46, 174]

*PDAC* pancreatic ductal adenocarcinoma, *PD-1* programmed cell death 1, *PD-L1* programmed cell death ligand 1, *PNI* perineural invasion.

Pancreatic ductal adenocarcinoma (PDAC) accounts for 90% of pancreatic cancers and is a highly malignant solid tumor characterized by an insidious onset, strong invasiveness, and a high recurrence or metastasis rate [48]. The standard treatment for PDAC involves a combination of surgery, chemotherapy, and radiation therapy, with the choice of therapy dependent on the stage and location of the cancer, as well as the overall health of the patient. Immune checkpoint inhibitors, such as nivolumab and pembrolizumab, have been shown to increase overall survival in some patients with advanced-stage disease [49]. However, due to a lack of effective screening methods, 80% of patients with PDAC are at an advanced stage when diagnosed, losing the chance of receiving resectable surgery. Resistance to chemotherapy and radiation therapy remains a great challenge to the treatment of PDAC. These factors contribute to the poor prognosis of PDAC patients, with an overall 5-year survival rate of only around 10% in 2021 [48]. Therefore, novel targets and therapies are required to enhance the outcome of PDAC.

Perineural invasion (PNI) is a typical characteristic of PDAC, defined as a tumor near the nerve where the tumor cells are located in at least 33% of the nerve circumference or any of the three layers of the nerve sheath [50]. PNI has been reported in about 80%–100% of patients with PDAC and is associated with postoperative recurrence and metastasis [51–54]. Meanwhile, tumor cells can produce and release neurotrophic factors like nerve growth factor (NGF) or brain-derived neurotrophic factor to promote tumor innervation [1, 55–57]. In the LSL-Kras^G12D/+^, LSL-Trp53^R172H/+^, Pdx-1-Cre (KPC) mouse model of PDAC, the number of sympathetic nerve fibers is tripled, and the number and density of calcitonin gene-related peptide (CGRP)-positive sensory nerves is increased by five times [58]. Analysis of clinical samples from patients with PDAC also illustrates a negative correlation between the density of nerve fibers in the tumor and survival [59]. Deep exploration of the interaction between nerve and tumor cells could lead to the identification of novel strategies for the treatment of PDAC.

In this review, we exhibit the mechanisms of PNI in PDAC and the role of different types of nerves innervating PDAC in tumor progression, summarize the potential mechanisms underlying the neuronal-cancer interaction, and discuss the current and potential therapeutic possibilities for PDAC.

## Innervation of Normal Pancreas and Pancreatitis

The pancreas receives both autonomic and sensory innervation. The autonomic innervation consists of sympathetic and parasympathetic nerves. The sympathetic nerves permeate the pancreatic ganglion, vascular system, endocrine islets, ducts, and lymph nodes [60]. On the other hand, activation of the parasympathetic nervous system promotes the release of digestive enzymes and reduces glucose-triggered insulin secretion. In terms of sensory nerves, substance P (SP) and CGRP-positive nerve fibers are distributed throughout the exocrine tissues and most islets [61]. Myelinated sensory fibers along with thinly-myelinated and unmyelinated peptidergic sensory fibers are present in the parenchyma of the head, body, and tail of the pancreas. The relative density of these sensory fibers is highest in the head and decreases towards the tail. In contrast, the post-ganglionic sympathetic fibers are relatively evenly distributed throughout the parenchyma of the pancreas [62]. The presence of autonomic and sensory nerves in the pancreas is crucial for maintaining its normal functions. The sympathetic and parasympathetic nerves work in tandem to regulate digestive processes and insulin secretion. The sensory nerves play a role in detecting changes in the environment and transmitting information about the state of the pancreas to the central nervous system.

**Fig. 1:**
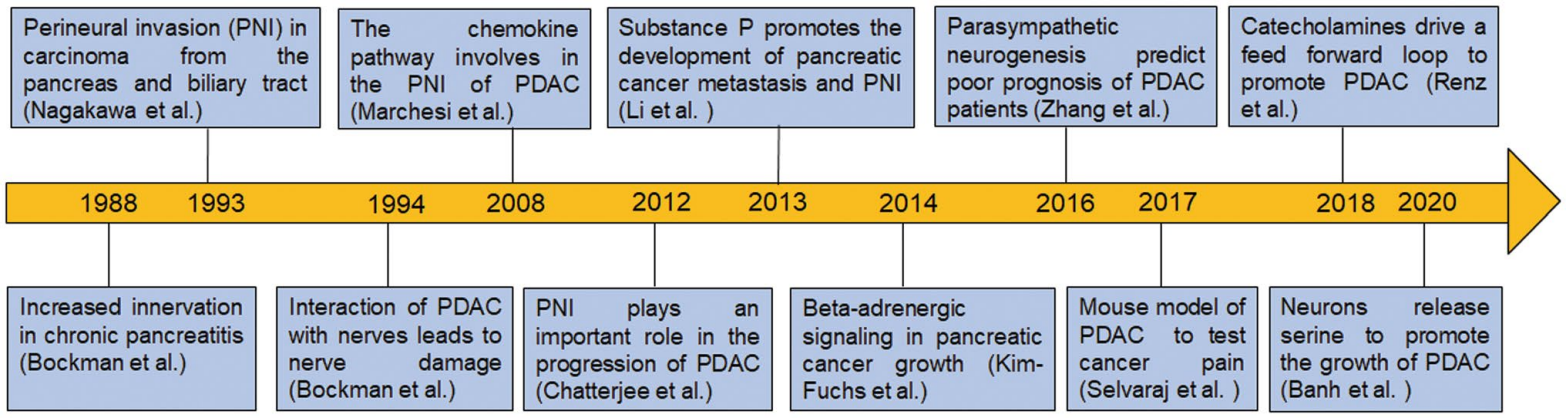
Timeline for major findings leading to the identification of crosstalk between peripheral innervation and PDAC. Abbreviations: PDAC, pancreatic ductal adenocarcinoma; PNI, perineural invasion

The phenomenon of increased number and diameter of pancreatic nerve fibers was first discovered in individuals with chronic pancreatitis (CP) [63]. CP is considered to be a high-risk factor for PDAC and has been shown to play a crucial role in the progression of this disease [64] (Fig. 1). In studies conducted on adult mice with PDAC, researchers found that expression of the K-Ras (G12V) mutation did not result in a tumor unless the mice also had CP [65]. This highlights the importance of considering CP as a risk factor for PDAC. In addition, research has shown that the expression of NGF and its receptor tropomyosin receptor kinase A (TrkA) is significantly higher in individuals with CP than in those with a normal pancreas. The increased expression of NGF is higher in metaplastic ductal cells and acinar cells that have dedifferentiated into tubular structures [66]. Further reports demonstrated that actively growing nerves in CP are associated with an activated NGF/TrkA pathway and a pain syndrome [66, 67]. As the normal pancreas with CP and intraepithelial neoplasia progresses into PDAC, pancreatic innervation is constantly remodeled and plays a crucial role in the worsening of the malignancy. The size (nerve hypertrophy) and number (nerve density) of pancreatic nerves are increased, the proportion of autonomic nerve fibers and sensory nerve fibers is altered (nerve remodeling), and there is infiltration of inflammatory cells around the nerve (pancreatic neuritis) or by PDAC cells (PNI) [51, 63, 68–73].

## Perineural Invasion (PNI) in PDAC

### General Background

PNI is a process in which cancer cells invade and spread along peripheral nerves. This histological characteristic has been found in a variety of tumors, including cancers of the head and neck, prostate, tongue, and pancreas. PNI involves a complex interplay between nerves and various cell types present in the TME, including Schwann cells, macrophages, and cancer-associated fibroblasts [74, 75]. Tumor cells interact closely with nerve components by releasing neurotrophic factors or exosomes and produce perineural niches, which provide a favorable environment for their survival and invasion and in turn, trigger the growth of nerves and stimulate the development of neural progenitor cells [76]. The underlying molecular mechanisms of PNI are governed by various factors such as NGF, glial cell line-derived neurotrophic factor (GDNF), and their corresponding receptors [77]. The cancer cells can cause damage to the neuronal sheath, activating nociceptive nerve fibers as a result of cancer-secreted mediators or stimuli from the extracellular matrix. This leads to the release of pro-inflammatory neuropeptides from peripheral nerve endings, further enhancing the spread of the tumor and causing pain. PNI has been demonstrated to be an independent predictor of poor prognosis among patients with oral squamous cell carcinomas, and nerve-tumor distance is a sensitive criterion to reclassify PNI [78]. A meta-analysis has shown that the presence of PNI is associated with a higher risk of biochemical recurrence of prostate cancer after radical prostatectomy or radiotherapy [79]. PNI is also an independent risk factor affecting the poor prognosis of patients with gastric cancer and colorectal cancer [80, 81].

### Mechanisms of PNI in PDAC

**Fig. 2:**
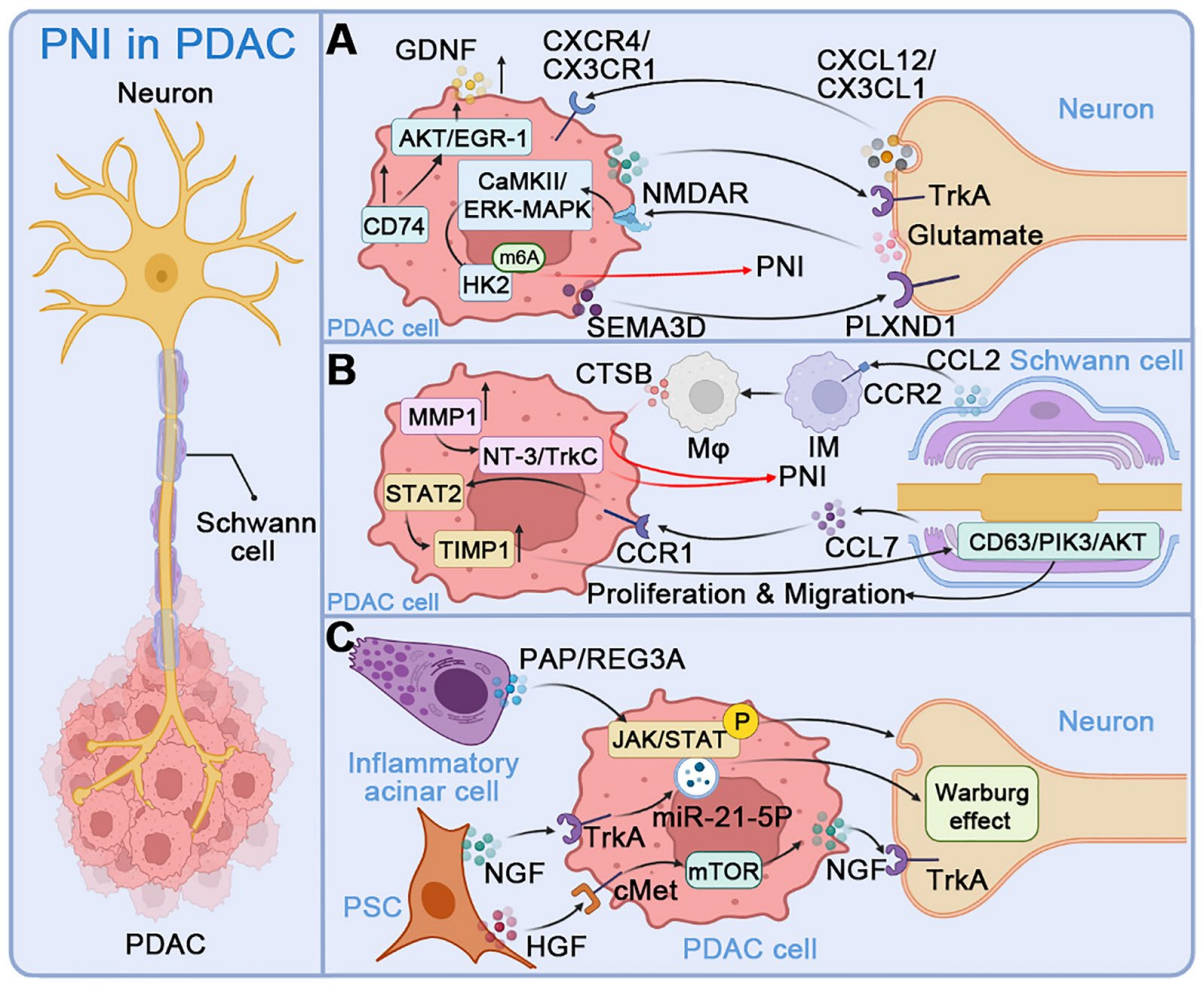
PNI in PDAC. **A** Direct interaction between PDAC cells and neurons in PNI. The neurogenic chemokines CXCL12 and CX3CL1 promote tumor cell invasion of nerves through their respective receptors CXCR4 and CX3CR1. Glutamate from neurons up-regulates HK2 expression through NMDAR2B and mRNA m6A modification of downstream Ca^2+^-dependent CaMKII/ERK-MAPK pathways, enhances glycolysis in nerve cells, and ultimately promotes PNI. Tumor cell-derived SEMA3D activates PLXND1 on DRG neurons to increase the migration and invasive activity of PDAC. The upregulation of CD74 on PDAC enhances its invasive capability and GDNF secretion *via* the AKT/EGR-1 pathway, thereby enhancing PNI. **B** Communication between PDAC cells and Schwann cells in PNI. The chemokine CCL7 produced by Schwann cells enhances the migration, invasion, and TIMP1 expression of PDAC cells through the CCR1/STAT2 pathway, and TIMP1 further stimulates the proliferation and migration of Schwann cells *via* the CD63/PI3K/AKT signal. CCL2 from Schwann cells drives CCR2-expressing IMs to differentiate into macrophages and enhance neural invasion through CTSB-mediated processes. In addition, high expression of MMP1 in PDAC promotes Schwann cell differentiation by stimulating the NT-3/TrkC signaling pathway. **C** Role of PSCs and acinar cells in PNI. PSCs induce PDAC cells to produce miR-21-5P exosomes through the NGF-TrkA axis, which further augments the Warburg effect of neurons and promotes PNI. HGF from PSCs activates the mTOR-NGF pathway through the c-Met receptor on PDAC cells, which boosts PNI. The pancreatitis-associated protein (PAP/REG3A) produced by inflammatory acinar cells in the microenvironment around the tumor promotes PNI by activating the JAK/STAT signaling pathway in PDAC. Abbreviations: PDAC, pancreatic ductal adenocarcinoma; NGF, nerve growth factor; Trk, tropomyosin receptor kinase; HK2, hexokinase 2; NMDAR2B, N-methyl-d-aspartate receptor subunit 2B; HGF, hepatocyte growth factor; IM, inflammatory monocytes; CTSB, cathepsin B; MMP, matrix metalloproteinase; PLXND1, Plexin D1; SEMA3D, Semaphorin 3D; GDNF, glial cell line-derived neurotrophic factor; PNI, perineural invasion; TIMP1, tissue inhibitor of metalloproteinases 1; PSC, pancreatic stellate cells; PAP, pancreatitis-associated protein; REG3A, regenerating islet-derived protein 3 alpha

Pancreatic cancer cells can reach the peripheral nerve at a short distance, which is the anatomical basis for why pancreatic cancer is prone to PNI. PDAC has a distinctive chronic inflammatory microenvironment that triggers the abnormal growth and malignant transformation of pancreatic cells. Chemokines, significant components of this environment, are known to contribute to both local invasion and distant metastasis of tumor cells [82]. Among them, CX3CL1 is a transmembrane chemokine highly expressed by numerous neurons, and it mediates the adhesion of endothelial cells to peripheral nerves. The overexpression of its receptor CX3CR1 in PDAC is associated with PNI and early postoperative recurrence [83]. PDAC cells can migrate to nerves that express CX3CL1 ligands by activating Gi protein and adhesion molecules [84]. The CXCL12/CXCR4 axis, another widespread chemokine signaling pathway, also plays a critical role in the tumor-matrix interaction and the neural infiltration of PDAC [85]. Aside from chemokines, Semaphorin 3D (SEMA3D) from tumor cells activates Plexin D1 (PLXND1) on dorsal root ganglion (DRG) neurons to increase the migration and invasion activity of pancreatic cancer cells. Increased expression levels of SEMA3D and PLXND1 have been confirmed in human PDAC specimens associated with PNI [86]. Nerve-derived glutamate also upregulates hexokinase 2 expression through mRNA m6A modification *via* N-methyl-d-aspartate receptor subunit 2B and the downstream Ca^2+^ pathway and ultimately promotes PNI [87] (Fig. 2A).

Schwann cells interacting with PDAC cells engage in the occurrence and development of PNI. The paracrine NGF of tumor cells activates Schwann cell autophagy, enhances the chemical attraction to tumor cells, and accelerates the removal and phagocytosis of myelin debris to promote early axonal and myelin regeneration [88, 89]. CCL7 secreted by Schwann cells enhances the migration, invasion, and tissue inhibitor of metalloproteinases 1 (TIMP1) expression of PDAC cells through the CCR1/STAT2 pathway, and TIMP1 further promotes Schwann cell proliferation and migration through CD63/PI3K/AKT signaling [90]. In addition, high expression of matrix metalloproteinase (MMP)1 in PDAC promotes the epithelial-mesenchymal transition and Schwann cell differentiation by stimulating the NT-3/TrkC signaling pathway [91]. CCL2 released by Schwann cells drives CCR2-expressing inflammatory monocytes (IM), preferentially recruiting them to the PNI site, where they differentiate into macrophages and enhance neural invasion through cathepsin B (CTSB)-mediated processes [92] (Fig. 2B).

Other cell types overexpressing NGF and GDNF like stromal pancreatic stellate cells (PSCs) or acinar cells in the TME also contribute to the PNI. The tumor-derived exosome miR-21-5p stimulated by NGF from PSCs activates the Warburg effect in neurons, upregulates the expression of nociceptor genes, and promotes the PNI [93]. Hepatocyte growth factor (HGF) produced by PSCs binds to the receptor c-Met on PDAC cells and endothelial cells [94] and activates the mTOR/NGF axis to boost PNI [95]. The up-regulation of CD74 enhances the migration and invasion of PDAC cells and promotes the production of GDNF through the AKT/EGR-1/GDNF axis to promote neural plasticity [96]. Moreover, the inflammatory acinar cells within the pancreas contribute to PNI through the production of pancreatitis-associated protein (pancreatitis-associated protein/regenerating islet-derived protein 3 alpha) (PAP/REG3A), which activates the JAK/STAT signaling pathway in cancer cells [86] (Fig. 2C).

### Clinical Significance

The occurrence of PNI in PDAC ranges from 70% to 95%, making it one of the most common features of these patients [51]. In patients with resectable PDAC, PNI represents a major determinant of tumor recurrence and post-operative survival, particularly in the early stages, where the invasion of nerves by cancer cells plays a driving role in disease progression [53]. In the setting of pre-operative gemcitabine-based chemoradiation therapy, PNI in resected PDAC specimens is significantly associated with disease-free survival and predicts the pattern of recurrence [97]. A meta-analysis of fourteen studies concluded that pre-operative PNI is also a promising marker for the prognosis of PDAC patients who undergo curative resection without neoadjuvant treatment [98]. Taken together, PNI in PDAC is an important prognostic factor, and early detection and management of PNI may help to improve clinical outcomes and survival in these patients.

## Sympathetic Innervation in PDAC

### General Background

The role of sympathetic innervation in various cancer types has been extensively investigated. Sympathetic activation increases the growth of primary tumors and elicits relevant symptoms, and tumor cells spread to normal adjacent tissues through adrenergic signaling pathways [46, 99, 100]. All β1, β2, and β3 adrenergic receptors are expressed in peripheral blood monocytes, activated T cells, monocytes, and monocyte-induced dendritic cells, and combinatorial sympathetic and cytotoxic T-lymphocyte-associated protein 4 (CTLA-4) blockade can inhibit the growth of murine melanoma [101]. Analysis of tumor samples from mice and patients shows an increase in the density of infiltrating autonomic nerve fibers [102], and that the autonomic innervation in the prostate regulates the development and spread of prostate cancer [17]. Specifically, sympathetic nerve fiber density is significantly higher in prostate tumors than in normal para-tumor tissue [102], and activation of sympathetic adrenalin signals is necessary for the early stages of prostate cancer and the initiation of the angiogenic switch. Angiogenesis is inhibited when the loss of β-adrenergic receptor signaling increases the oxidative phosphorylation of endothelial cells by increasing the expression of mitochondrial cytochrome c oxidase assembly factor 6 [20]. In addition, the adrenergic signal is closely associated with the malignant invasion of the tumor [102]. The adrenergic signal up-regulates the expression of CCL2 in lung stromal cells before metastasis, increases the infiltration of monocytes and macrophages into lung tissue, and promotes the colonization of tumor cells through lung metastasis [23].

### Mechanisms in PDAC

**Fig. 3:**
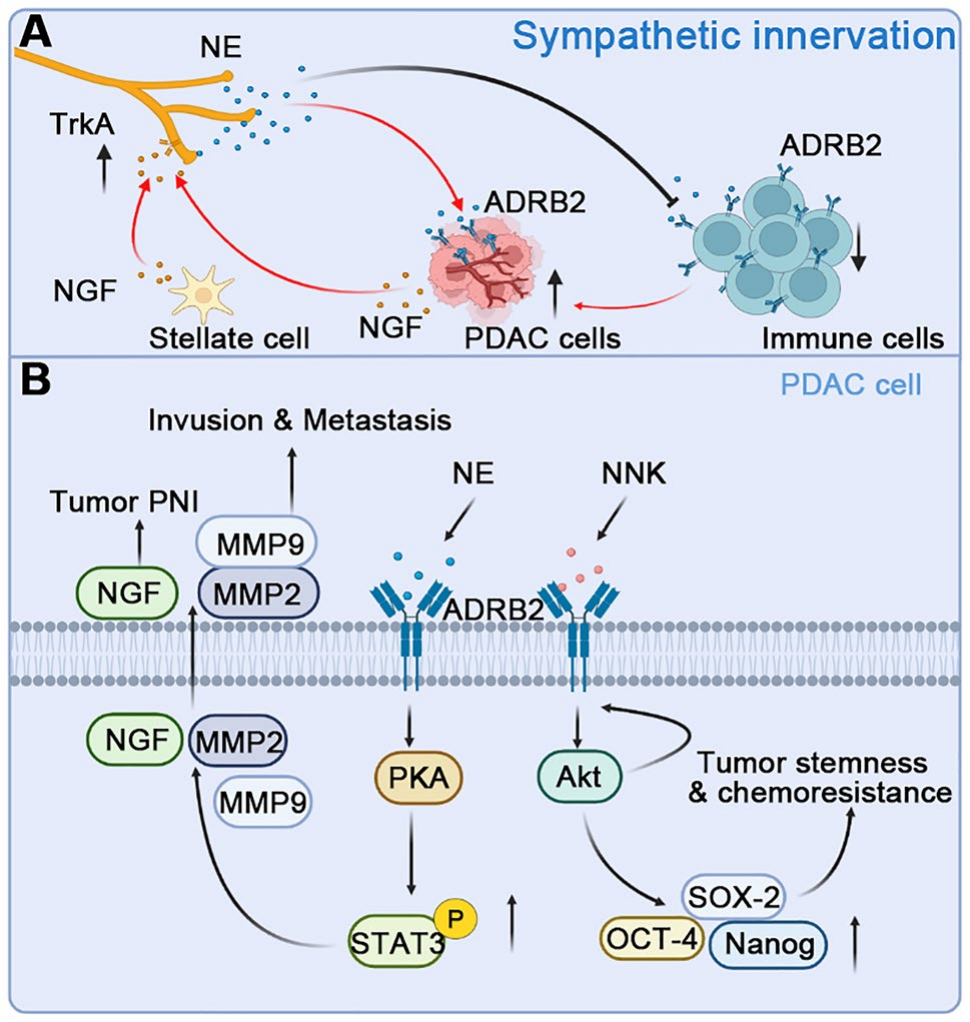
Sympathetic innervation and signaling pathways between NE and PDAC cells. **A** PDAC cells and pancreatic stellate cells release NGF to act on the TrkA receptors on the sympathetic nerve and promote the sympathetic innervation of the tumor. On the contrary, the neurotransmitter NE released by the sympathetic nerve promotes the proliferation of tumor cells through the ADRB2 receptors on tumor cells and endothelial cells and inhibits immune function by binding to receptors on immune cells to promote tumor progression. **B** The combination of NE and ADRB2 activates the downstream PKA-STAT3 signaling pathway, increases the phosphorylation level of STAT3, and promotes the release of NGF, MMP2, and MMP9 from tumor cells. Activation of ADRB2 by NNK triggers the downstream Akt pathway which in turn increases the expression of ADRB2. The levels of OCT-4, SOX-2, and Nanog are also up-regulated and thus promote the tumor stemness and chemoresistance of PDAC. Abbreviations: PDAC, pancreatic ductal adenocarcinoma; NE, norepinephrine; NGF, nerve growth factor; TrkA, tropomyosin receptor kinase A; ADRB, β-adrenergic receptor; MMP, matrix metalloproteinase; NNK, 4-(methylnitrosamino)-1-(3-pyridyl)-1-butanone; OCT-4, octamer-binding transcription factor-4; SOX-2, SRY-Box transcription factor 2

The pancreas is innervated by sympathetic nerve fibers that release both adrenergic and neurotrophic factors which drive the cancer-nerve feedforward loop [100]. Tumor-derived neurotrophic factors bind to corresponding receptors like TrkA on sympathetic nerves, induce neurogenesis and axonogenesis [103, 104], and thus increase cancerous innervation [51, 57, 100]. The secretion of neurotrophic factors or induction of tumor cell-derived exosomes by PSCs also potentiates nerve proliferation and increases tumor innervation [104–106] (Fig. 3A). Catecholamines like norepinephrine (NE) from sympathetic nerves act on ADRB2 from PDAC cells and promotes their PNI, invasion, and metastasis *via* the activation of the ADRB2/PKA/STAT3 signaling pathway, which increases the production of NGF and MMP2/9 [107]. Meanwhile, the ADRB2-Akt pathway in PDAC activated by 4-(methylnitrosamino)-1-(3-pyridyl)-1-butanone (NNK) mediates smoking-induced tumor stemness and gemcitabine resistance by increasing octamer-binding transcription factor-4 (OCT-4), SRY-Box transcription factor 2 (SOX-2), and Nanog in pancreatic cancer cells [108] (Fig.3B). The orthotopic mouse model of breast cancer shows negligible effects of circulating epinephrine on β2-adrenergic signaling [109]. In addition to acting directly on cancer cells, sympathetic nerves have also been shown to regulate the immune function of tumor-infiltrating lymphocytes through ADRB2 on CD8^+^T cells in melanoma [101]. The density of PD-L1^+^ tumor-associated nerves is inversely correlated with that of CD8^+^ tumor-associated lymphocytes and predicts higher biochemical recurrence [110].

### Therapeutic Implications

Since sympathetic activation in TME leads to the progression of PDAC, blocking the adrenergic signaling pathway may be a potential therapeutic strategy. Pharmacological ablation of sympathetic nerves by 6-hydroxydopamine [5, 23] results in an increased proportion of neutrophils in the spleen of infected and uninfected mice, suggesting that sympathetic nerves may also be involved in the inhibition of neutrophil infiltration during infection [111]. In addition, the application of adrenaline-signaling pathway blockers [112, 113] such as propranolol [23, 114] can reverse the effect of chronic stress on the progression of PDAC [46]. When combined with gemcitabine, it reduces NGF expression and nerve density and improves the survival rate of KPC mice [100]. Combined sympathetic and CTLA-4 blockade inhibits murine melanoma growth by targeting infiltrating T cells [101]. Other drugs that target adrenergic signals including antipsychotics and tricyclic antidepressants have been shown to reduce the risk of colorectal cancer and glioma and are associated with increased survival [115–120].

## Parasympathetic Innervation in PDAC

### General Background

In many solid tumors, parasympathetic input is provided by the vagus nerve, which has been shown to modulate tumor growth in an organ-specific way. The stomach is innervated predominantly by the parasympathetic nervous system, where choline can stimulate the gastric epithelium to overexpress NGF, which leads to further enlargement of the enteric nerve and promotes canceration [39]. Acetylcholine can also promote the self-renewal and immune escape of CD133^+^ thyroid cancer cells through activation of the CD133/PI3K/Akt pathway [14]. In human prostate cancer cell lines and mouse models of prostate cancer, cholinergic signals are transduced in the tumor stroma through the muscarinic cholinergic receptor 1 (CHRM1) to promote tumor invasion [121]. The ability of muscarinic agonists to stimulate growth and muscarinic receptor antagonists to inhibit tumor growth has also been demonstrated for breast, melanoma, lung, colon, ovarian, and brain cancer [122].

### Mechanisms in PDAC

**Fig. 4:**
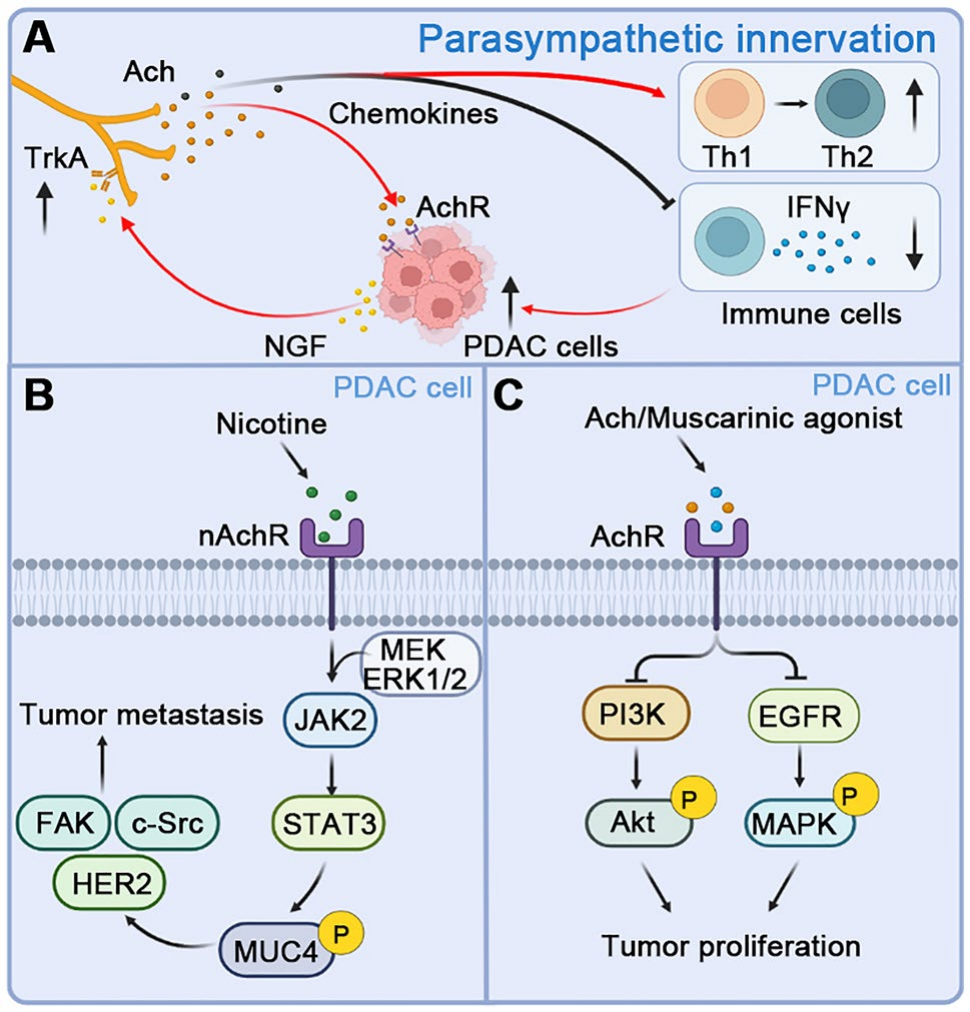
Parasympathetic innervation and crosstalk with PDAC cells. **A** PDAC cells release the neurotrophic factor NGF, which combines with TrkA on the parasympathetic nerve and promotes the proliferation of the parasympathetic nerve and the innervation of PDAC, resulting in an increase in the level of acetylcholine (ACh) and promotes the growth of PDAC cells. In addition, parasympathetic nerves can also promote the transformation of Th1 to Th2 immune cells by releasing chemokines and inhibiting the release of IFNγ from CD8 ^+^ T cells, resulting in immunosuppression. **B** The activation of the α7 subunit of nAChRs by nicotine increases the expression of MUC4 through JAK2/STAT3 downstream signaling and in cooperation with the MEK/ERK1/2 pathway. MUC4 upregulation further promotes the metastasis of PDAC *via* the activation of downstream effectors, such as HER2, c-Src, and FAK. **C** Activation of ACh receptors by muscarinic agonists inhibits downstream EGFR/MAPK and PI3K/AKT signaling pathways and inhibits the proliferation of PDAC cells. Abbreviations: PDAC, pancreatic ductal adenocarcinoma; NGF, nerve growth factor; TrkA, tropomyosin receptor kinase A; nAChR, nicotinic acetylcholine receptor; ACh, acetylcholine

In PDAC, over-expressed parasympathetic and cholinergic receptors have been detected in tumor tissue from patient and mouse models [123, 124]. Patients with PDAC and high parasympathetic density showed higher tumor budding and earlier recurrence rates than patients with low parasympathetic density [123]. The cholinergic signal enhances tumor growth by inhibiting the T cell response in the orthotopic PDAC model. When the parasympathetic nerve is stimulated, acetylcholine is released from the postganglionic fibers. Acetylcholine inhibits the recruitment of CD8^+^ T cell infiltration to PDAC through histone deacetylase 1-mediated CCL1, and directly inhibits CD8^+^ T-cell production of IFNγ in a concentration-dependent manner, reducing the Th1/Th2 ratio in the TME. In contrast, in tumor-bearing mice, vagotomy blockade not only reduces PNI but also increases CD8^+^ T cell infiltration and mouse survival [125] (Fig. 4A). Nicotine also promotes the metastasis of pancreatic cancer *via* the activation of the nicotinic acetylcholine receptor/JAK2 /STAT3 downstream signaling cascade and the upregulation of MUC4 expression [126] (Fig. 4B). However, Renz and colleagues showed that subdiaphragmatic vagotomy accelerates tumorigenesis and a muscarinic agonist suppresses tumorigenesis *via* MAPK and PI3K/AKT signaling [127] (Fig. 4C), suggesting that parasympathetic innervation may play distinct roles during the initiative and progressive stages of PDAC.

### Therapeutic Implications

Blocking parasympathetic innervation with bilateral subdiaphragmatic vagotomy improves the survival of PDAC mice [47]. Similarly, abrogation of cholinergic input by vagotomy or chemical denervation inhibits the growth of gastric cancer by blocking the M3 receptor-mediated Wnt pathway [39]. It also enhances the therapeutic effect of systemic chemotherapy and prolongs survival. The inhibitory effect induced by denervation is related to the inhibition of Wnt signaling and stem cell expansion [128]. Carbachol is a selective CHRM3 agonist, which enhances prostate cancer growth *via* the CaM/CaMKK-mediated phosphorylation of Akt. Blocking CHRM3 by darifenacin treatment inhibits prostate cancer growth and castration resistance *in vitro* and *in vivo* [129]. In this line, other studies have also reported that CHRM1 is involved in regulating the migration and invasion of prostate cancer through the Hedgehog signaling pathway. The selective CHRM1 antagonist pirenzepine inhibits the migration and invasion of cancer cells [121]. Furthermore, the application of the CHRM inhibitors Pirenzepine [17] and Darifenacin [129] reduces migration and invasion, thereby suppressing cancer cell proliferation.

## Sensory Innervation in PDAC

### General Background

The role and mechanism of sensory innervation in tumor progression have been increasingly investigated recently. In head and neck cancer, loss of tumor protein 53 leads to adrenergic transdifferentiation of tumor-associated sensory nerves through loss of the microRNA miR-34a, and tumor growth is suppressed by sensory denervation [130]. Melanoma cells interact with nociceptive sensory neurons, leading to increases in their neurite outgrowth and release of CGRP, which may further increase the exhaustion of cytotoxic CD8^+^ T cells and promote tumor immune escape [131]. In oral mucosa carcinomas, the low-glucose environment drives the production of NGF, which may further promote the release of CGRP from nociceptive nerves. CGRP subsequently induces cytoprotective autophagy in cancer cells that thrive in nutrient-poor environments [132]. CGRP is also an important neurotransmitter in the neural-immune axis, negatively regulating the infection-related immune response [133–135]. In CGRP-knockout mice with oral squamous cell carcinoma, the tumor burden is significantly reduced with increased tumor-infiltrating lymphocytes [29].

### Mechanisms in PDAC

**Fig. 5:**
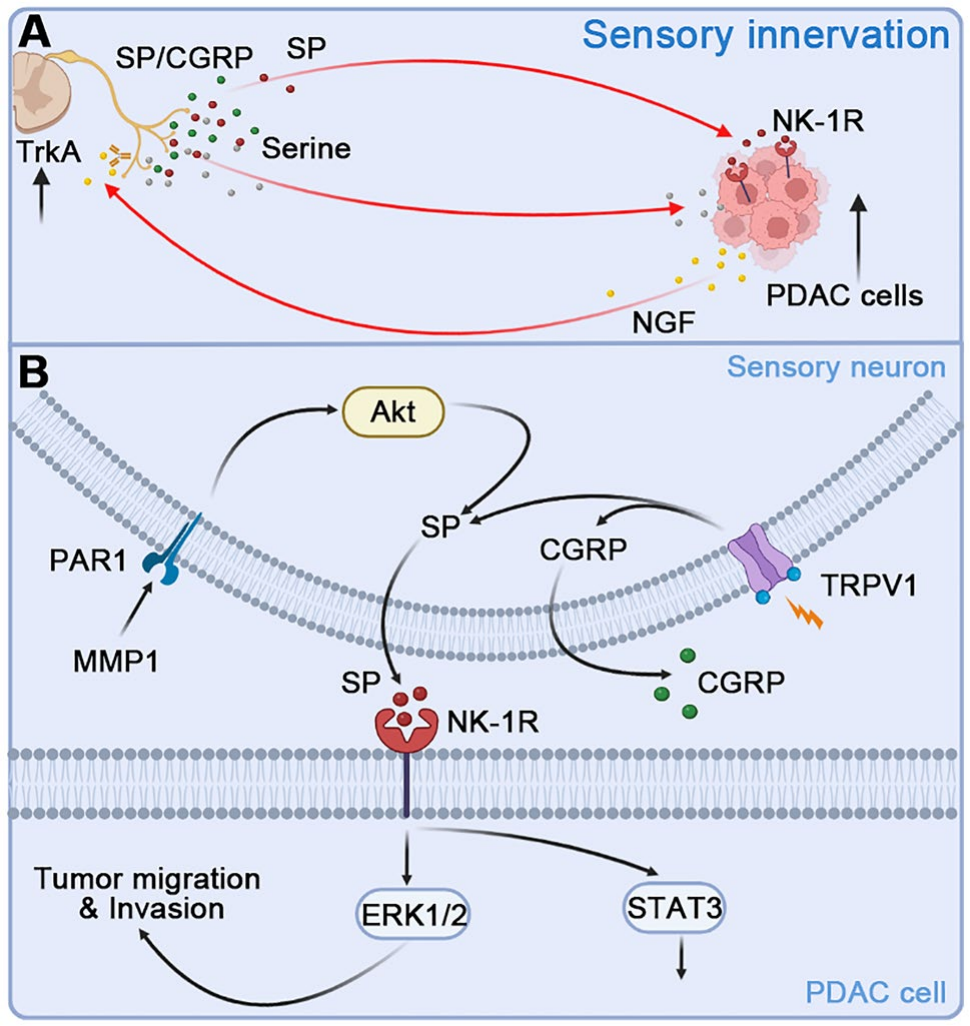
Molecular mechanisms by which sensory neurons promote PDAC progression. **A** PDAC cells release NGF, promote the sprouting of sensory nerves *via* TrKA, resulting in increased levels of CGRP and SP, and promote the growth of PDAC cells by binding to the SP receptors NK-1Rs on tumor cells. Sensory nerves also secret exogenous serine to maintain the survival of PDAC. **B** TRPV1 is activated by the acidic environment of TME, resulting in the increasing release of SP and CGRP from nociceptive neurons. MMP1 binding to its receptor PAR1 in DRG neurons mediates PNI of PDAC cells by activating the Akt pathway and induces the release of SP. SP promotes the migration, invasion, and PNI of PDAC cells through NK-1Rs by the activation of downstream ERK signaling. It also fuels the progress of PanIN by activating the STAT3 signaling pathway. Abbreviations: PDAC, pancreatic ductal adenocarcinoma; NGF, nerve growth factor; TrkA, tropomyosin receptor kinase A; NK-1R, neurokinin 1 receptor; CGRP, calcitonin gene-related peptide; SP, substance P; TME**,** tumor microenvironment; TRPV1, transient receptor potential vanilla 1; PAR1, protease-activated receptor-1; MMP, matrix metalloproteinase; PanIN, pancreatic intraepithelial neoplasms. PNI, perineural invasion

Neurotrophic factors derived from PDAC cells can induce the proliferation of nerve fibers including sensory nerves. In turn, sensory nerves promote the migration and invasion of cancer cells *in vitro* and *in vivo* by releasing neurotrophic factors or chemokines [58, 86, 136]. In the nutrient-poor microenvironment of PDAC, the sprouting sensory nerve could also secret exogenous serine to maintain the survival of cancer cells [103] (Fig. 5A). In PDAC patient samples, high expression of neurotrophic factors has been confirmed to be associated with PNI [86]. Transient receptor potential vanilla 1 (TRPV1) is an ion channel expressed on nociceptive sensory neurons and mediates thermal pain. TRPV1 can be activated by the acidic environment of the TME [137], resulting in increased release of SP and CGRP from nociceptive neurons. In the early stage of primary PDAC formation, MMP1 induces protease-activated receptor-1 (PAR1) expression in DRGs to release SP by activating the AKT pathway, thereby activating PDAC cells expressing neurokinin 1 receptor (NK-1R) and enhancing cell migration, invasion, and PNI through the SP/NK1R/ERK signal. In addition, SP can also induce the expression of MMP2 in tumor cells [138, 139]. Organoid culture experiments have also confirmed that sensory neurons promote the proliferation of pancreatic intraepithelial neoplasms (PanIN)-like organs through SP-NK1-R signaling and STAT3 activation. In the genetically engineered mouse model of PDAC, sensory denervation leads to a loss of STAT3 activation and slows down the progression of PanIN to tumors [140] (Fig. 5B).

### Therapeutic Implications

Drugs targeting nociceptor nerves, neuropeptides, and their receptor pathways are mainly used for pain treatment. But they now appear to have great potential in treating cancer. In acute myeloid leukemia and Ewing sarcoma, the efficacy of some drugs targeting CGRP and its receptors calcitonin receptor-like receptor (CALCRL) and receptor activity-modifying protein 1 (RAMP1) has been verified. The CGRP antagonist olcegepant increases differentiation and reduces the burden of leukemia and key stem cell characteristics in mouse models of acute myeloid leukemia, while small molecule inhibitors targeting CGRP receptors reduce the growth of Ewing sarcoma [141–143]. Also, TRPV1 is desensitized by capsaicin, and capsaicin or resiniferatoxin has been used as an alternative pharmacological method to block pain by depleting CGRP and SP without stimulation or toxicity. In addition, intravesical injection of resiniferatoxin improves bladder function in patients with an overactive bladder. In the bone cancer model, intrathecal injection of resiniferatoxin effectively relieves pain and improves function without significant long-term side-effects. These suggest the multiple therapeutic effects of targeting sensory nerves [15, 44, 144–150].

## Pain Relief Targeting the Nerves Innervating PDAC

Cancer cells communicate with their surrounding environment [151]. Non-tumor cells in the TME may directly or indirectly interact with cancer cells, affecting the proliferation, migration, invasion, or drug resistance of PDAC. Evidence shows that sympathetic, parasympathetic, and sensory nerves undergo different forms of neuronal remodeling during the development of normal pancreatic tissue into PDAC. This has been confirmed in animal experiments and clinical pathological samples. Interstitial components such as nerve fibers in the TME play a direct or indirect role in promoting neurogenesis and tumor growth through various neurotransmitters, neurotrophic factors, and chemokines. The neural supply of amino-acids (such as serine) to the nutritionally deficient TME is also an important factor in the progression of PDAC [103]. Therefore, targeting nerves may be a promising strategy to treat cancer and immune evasion in the TME [152].

Pain is one of the common clinical symptoms of advanced PDAC. The abdominal pain symptoms can arise from various causes including tissue damage, inflammation, ductal obstruction and infiltration, and/or a direct mass effect on nerves in the celiac plexus [70]. At present, clinical treatments for pancreatic cancer pain mainly depend on opioids and surgery. Commonly-used analgesics are bucinnazine hydrochloride and morphine, but long-term use usually causes drug tolerance and adverse drug reactions. Surgical treatment can be categorized into celiac plexus neurolysis (CPN) and celiac ganglion neurolysis (CGN) [153–156], which are variations of an interventional technique for the diagnosis and treatment of concealed abdominal pain. Also, botulinum toxin is used as a preventive strategy for precancerous lesions and local treatment of low-risk tumors in prostate cancer, or as an adjunct to tumor treatment to reduce recurrence rates [157]. Neurolytic agents such as ethanol and phenol are used to permanently destroy the celiac plexus. Local anesthetics, most commonly bupivacaine or lidocaine, are used in combination with steroids and ethanol for the sake of reducing pain and the usage of painkillers [158, 159]. However, short-term back pain may occur at the injection site within 72 hours after celiac nerve block [156]. Other common side-effects include postural hypotension and diarrhea, which may be related to blocking or damaging sympathetic signals. Severe postoperative complications include lower limb paralysis and multiple organ failure, pain, and loss of temperature sensation. Other cases have been reported in which celiac trunk thrombosis after celiac artery spasm causes liver and spleen infarction, as well as stomach and proximal small intestine infarction [160]. In a prospective study of patients with unresectable PDAC and abdominal pain, compared with CPN, CGN shortened the median survival time and did not improve pain, quality of life, or frequency of adverse events [161]. Therefore, celiac nerve block should be carefully considered.

To this end, safer and more effective treatments for PDAC-related pain are urgently needed. Deep exploration of cancer-nerve crosstalk may provide potential targets [162, 163], such as neurotransmitters, neurotrophic factors, and chemokines. The effectiveness and safety of these strategies have been verified in preclinical animal models. Drugs currently known to regulate sympathetic or parasympathetic signals, such as the selective or non-selective β-blocker propranolol or metoprolol, or parasympathetic-like drugs, tend to have an antinociceptive effect with promising suppression of PDAC progression [164]. In turn, lidocaine or bupivacaine treatment has proved effective in inhibiting tumor growth and nerve fiber formation as well as cancer pain relief [165, 166]. Similarly, targeted neurotrophic factor therapy has also demonstrated tumor-suppressive effects in triple-negative breast cancer [167]. However, differences in cholinergic responses between cancers such as gastric and pancreatic cancers need to be carefully identified. In addition, capsaicin or resiniferatoxin targeting nociceptor sensory nerves could reduce the production of CGRP and SP, thus inhibiting PDAC growth and attenuating cancer pain. In addition to existing methods, recently developed neural engineering techniques allow the selective manipulation of the specific type of nerve fibers in the TME, in order to control the cancer progression and pain [152, 168].

## Conclusions and Perspectives

Here we highlight the crucial role of tumor-innervating nerves as key TME components regulating the initiation and progression of PDAC as well as other cancer types. In addition, sympathetic, parasympathetic, or sensory innervation modulates distinct signaling pathways of tumor survival or immune escape. Selective peripheral nerve blockade or abrogation, and drugs targeting neuropeptides and their receptor pathways may be promising treatments for PDAC and cancer pain. However, it remains unclear how sensory nerves regulate the infiltration and function of immunological components in the TME of PDAC. Moreover, the direct or indirect modulation of cancer cells, stromal cells, and immune cells by tumor innervation interacting as a network in the TME warrants specific identification and detailed illustration. Recently, innervated wild-type or KPC murine pancreatic organoids have been well established, providing an *ex vivo* model to further study pancreatic neuropathy [169]. Future research is also needed to determine optimal strategies for tumor innervation based on current findings and to explore potential synergistic benefits when combined with chemotherapy or immunotherapy.
